# A low-cost three-dimensional laser surface scanning approach for defining body segment parameters

**DOI:** 10.1177/0954411917727031

**Published:** 2017-08-17

**Authors:** Petros Pandis, Anthony MJ Bull

**Affiliations:** Department of Bioengineering, Imperial College London, London, UK

**Keywords:** Body segment parameters, anthropometrics, laser surface scanner, low-cost three-dimensional scanner, musculoskeletal models

## Abstract

Body segment parameters are used in many different applications in ergonomics as well as in dynamic modelling of the musculoskeletal system. Body segment parameters can be defined using different methods, including techniques that involve time-consuming manual measurements of the human body, used in conjunction with models or equations. In this study, a scanning technique for measuring subject-specific body segment parameters in an easy, fast, accurate and low-cost way was developed and validated. The scanner can obtain the body segment parameters in a single scanning operation, which takes between 8 and 10 s. The results obtained with the system show a standard deviation of 2.5% in volumetric measurements of the upper limb of a mannequin and 3.1% difference between scanning volume and actual volume. Finally, the maximum mean error for the moment of inertia by scanning a standard-sized homogeneous object was 2.2%. This study shows that a low-cost system can provide quick and accurate subject-specific body segment parameter estimates.

## Introduction

Body segment parameters (BSPs) are used in many different applications in ergonomics, but they can also be used in inverse dynamic modelling of the musculoskeletal system in which the human body is modelled as a linked-segment system. It has been shown that different BSP measurement techniques can affect musculoskeletal kinetic analysis by up to 20%.^1^

BSPs are quantified in different ways as follows: through the use of regression equations, geometrical modelling and direct measurement techniques, such as scanning technology. Regression equations are most commonly used with variables such as body mass (BM) and body height (BH) as the only input variables,^2^ where others include sex^3^ or race and age.^4–6^ Geometrical modelling techniques use a mathematical model of the human body based on experimentally determined distribution of mass and standard anthropometric dimensions of the subject. Finally, scanning technology includes various different medical imaging techniques, such as computed tomography (CT),^7,8^ magnetic resonance imaging (MRI),^9–11^ dual-energy X-ray (DEXA)^12^ and gamma-ray.^13^ Several limitations remain with these imaging techniques: they are time consuming, the facilities may not be readily available, the cost is high and, in some cases, there is exposure to ionising radiation. Other scanning technologies have more recently been proposed in the literature, including the re-purposing of gaming technologies,^14^ smart/mobile phones,^15^ photonic scanning^16,17^ and the use of multiple cameras.^18^ These methods have potential for use in musculoskeletal modelling; however, they have not been validated for the measurement of BSPs. The aim of this study was to devise, develop and test an easy, fast, accurate and low-cost scanning technique for measuring subject-specific BSPs.

## Materials and methods

### Equipment, software and calibration

**Figure 1.**
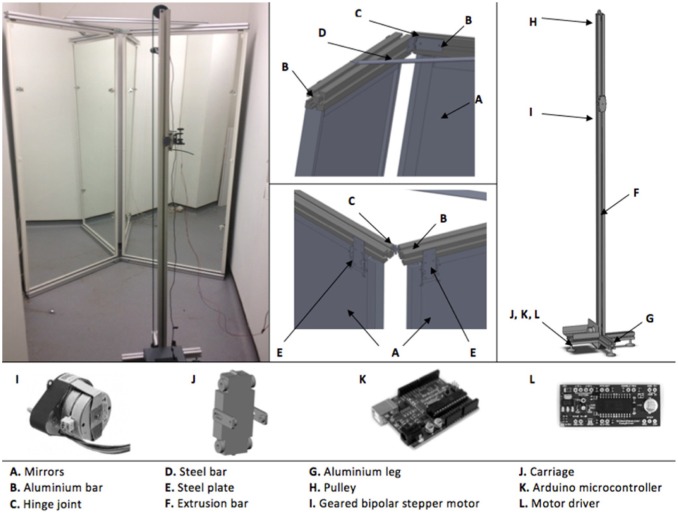
Laser scanner device structure.

The measurement system devised consists of a web camera, green laser on a linear drive actuator, mirror structure and a software system for data acquisition and processing. The mirror configuration comprises two mirrors (2220 mm × 914 mm × 40 mm) with a mounting frame and base plate. The linear drive actuator comprises a carriage for the laser mounted on a rail with a stepper motor and driver controlled with a single-board microcontroller (Figure 1). A LabVIEW (National Instruments Corporation, Austin, TX, USA) user interface was designed to calibrate the laser drive actuator, set the start and end point of movement and assign the speed of motion.

The software DAVID 2.1 (DAVID Vision Systems, Braunschweig, Germany) was used for three-dimensional (3D) data acquisition, image reconstruction and calibration.^19^ Modifications were made to actuate the laser driver and pre-calibrate the mirror setup, accounting for the offset and rotation between the left and right panels and the distance between the panel and the mirrors.

**Figure 2.**
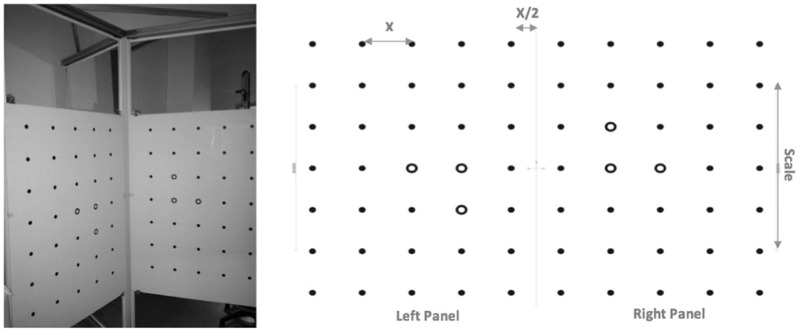
Setting up the camera’s calibration panels.

Two different-sized calibration panels were devised to quantify the location, view direction and focal length of the camera, where each panel consists of 70 markers (Figure 2). X is the distance between two markers (from centre to centre) in every direction (horizontal and vertical). The diameter of each marker cannot be the same as the distance X. The scale parameter is equal to four times the distance X. The distance of the inner rows from the cutting (or folding) edge is half the distance X. Note that hollow markers have to be set up as shown in Figure 2.

### Protocol

A mannequin was used to test the whole process of scanning, reconstructing and editing a 3D model and estimating the body segment volume. The mass is proportional to the volume for a uniform density; therefore, the volume has an indirect correlation to BSPs. In this case, the mannequin’s density is not uniform and so volume was used. The mannequin was scanned five times with focus on the right upper limb (without the hand). Each scan took between 8 and 10 s. The start and end points were defined to cover the size of the object, and the procedure took place in a dark room and the camera was mounted so that only the laser line was visible. Computer-aided design (CAD) software packages, SolidWorks 2011 (SolidWorks Corp., Concord, MA, USA) and Geomagic Studio 12 (Raindrop Geomagic Inc., Research Triangle Park, NC, USA), were used to edit the images (de-noising, smoothing and mesh merging). Finally, the volume of the mannequin’s upper limb was measured using a water displacement technique and buoyancy theory

B=ρ×V×g

where *B* is the buoyant force, *ρ* is the displaced fluid’s density in kg/m^3^, *V* is the displaced fluid volume in m^3^ and *g* is the gravitational acceleration. The arm mass was measured using scales and thereafter the arm was placed into a box full of water. The experimental procedure was repeated five times.

### Modelling and analysis

**Figure 3.**
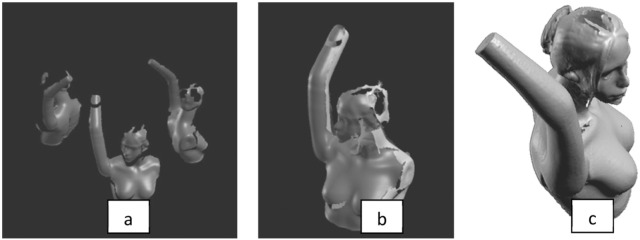
(a) De-noised and smoothed 3D mesh, (b) merged 3D meshes and (c) final 3D scan after editing in Geomagic Studio 12.

After scanning, the software computes the 3D model/mesh of the mannequin. This is then masked to remove background information, smoothed and de-noised prior to merging of the scans from the mirrors and saved as an .STL file (Figure 3).

In this study, the model was trimmed to include only the upper limb. Geomagic Studio 12 was used for filling the mesh holes and turning the 3D data into an accurate polygon and a native CAD model. Element reduction was performed in Geomagic Studio 12. The model was reduced from 18,000 to 12,000, 3600 and 2500 polygons. SolidWorks 2011 was used to create a solid model and thereafter to measure the volume of the arm for each number of polygons and assess the effect of element reduction. The scanning process was repeated five times, and the results were compared with the measured volume.

**Figure 4.**
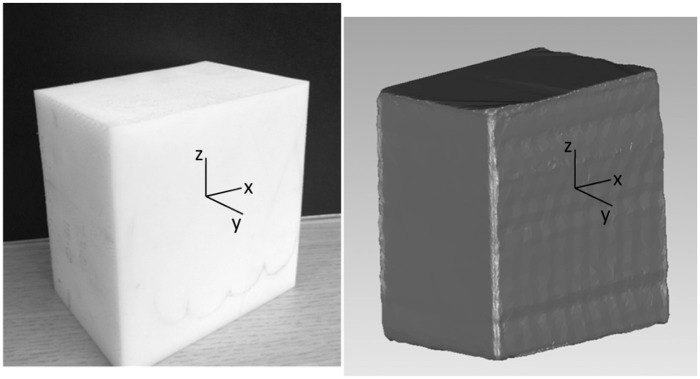
Experimental standard-sized homogeneous object.

A standard-sized homogeneous object of density of 1.15 g/cm^3^ was used to quantify BSPs. After scanning, SolidWorks 2011 was used for the automatic calculation of mass, moment of inertia and centre of mass (Figure 4). All data were distributed normally and two-tailed paired samples *t*-tests were used to assess differences.

## Results

Element reduction from 18,000 to 2500 polygons caused a reduction in measured volume of 0.000009 m^3^ (0.4%; Table 1).

**Table 1.** Effect of element reduction on measured volume.

No. of polygons	Volume (m^3^)	Difference (%)
2500	0.002148	−0.4
3671	0.002152	−0.2
12,000	0.002157	0
18,000	0.002157	

The scanning volume was measured to be 3.1% greater than for the buoyancy measures (Table 2). This was not statistically significant (*p* = 0.0779).

**Table 2.** Actual volume versus volume from the 3D models (2500 polygons).

	Measured volume using the buoyancy technique (m^3^)	Measured volume by laser scanning (m^3^)
	0.002031	0.002148
	0.002049	0.002168
	0.002028	0.002103
	0.002056	0.002030
	0.002055	0.002091
Average	0.0020438	0.0021080
SD	0.0000134	0.0000538
Difference	3.1% (*p* = 0.0779, paired samples two-tailed *t*-test)	

SD: standard deviation.

BSPs for the standard shape were all within 2.2% of the true values (Table 3).

**Table 3.** Standard object body segment parameters.

Properties	Actual	Measured (mean ± SD)	Mean error (%)
Mass (kg)	0.99665	1.00480 ± 0.02863	0.8
Moment of inertia *z* (kg m^2^)	0.00138	0.00142 ± 0.00003	2.2
Moment of inertia *x* (kg m^2^)	0.00148	0.00152 ± 0.00002	2.2
Moment of inertia *y* (kg m^2^)	0.00197	0.00198 ± 0.00002	0.2

## Discussion

In this study, a low-cost 3D scanner was developed and tested for use in the measurement of BSPs for ergonomic and musculoskeletal dynamic applications. The technology was able to scan an arm in less than 10 s, and a processing technique was developed using off-the-shelf software packages that allowed the rapid calculation of BSPs within an accuracy of ±2.2%. The new method has some limitations, including the requirement for manual intervention to define the ends of the body segments and the image processing steps that includes de-noising and mesh merging.

Body scanning has progressed rapidly in recent years and this is set to continue as gaming technologies become more ubiquitous. However, the requirements for body scanning for gaming are different to those for advanced ergonomics using musculoskeletal modelling in which errors in BSPs can produce high errors in the calculation of muscle and joint forces for high acceleration activities.

This study has shown that an inexpensive, fast-running scanning approach can be used to obtain BSPs for subsequent use in ergonomics or musculoskeletal modelling.
